# Uncovering the Tumor Antigen Landscape: What to Know about the Discovery Process

**DOI:** 10.3390/cancers12061660

**Published:** 2020-06-23

**Authors:** Sara Feola, Jacopo Chiaro, Beatriz Martins, Vincenzo Cerullo

**Affiliations:** 1 Drug Research Program (DRP) ImmunoViroTherapy Lab (IVT), Division of Pharmaceutical Biosciences, Faculty of Pharmacy, Viikinkaari 5E, University of Helsinki, 00790 Helsinki, Finland; sara.feola@helsinki.fi (S.F.); jacopo.chiaro@helsinki.fi (J.C.); beatriz.martins@helsinki.fi (B.M.); 2 Helsinki Institute of Life Science (HiLIFE), Fabianinkatu 33, University of Helsinki, 00710 Helsinki, Finland; 3 Translational Immunology Program (TRIMM), Faculty of Medicine, Haartmaninkatu 8, University of Helsinki, 00290 Helsinki, Finland; 4 Digital Precision Cancer Medicine Flagship (iCAN), University of Helsinki, FI-00014 Helsinki, Finland

**Keywords:** tumor antigens, immunopeptidome, cancer immunotherapy, epitope prediction

## Abstract

According to the latest available data, cancer is the second leading cause of death, highlighting the need for novel cancer therapeutic approaches. In this context, immunotherapy is emerging as a reliable first-line treatment for many cancers, particularly metastatic melanoma. Indeed, cancer immunotherapy has attracted great interest following the recent clinical approval of antibodies targeting immune checkpoint molecules, such as PD-1, PD-L1, and CTLA-4, that release the brakes of the immune system, thus reviving a field otherwise poorly explored. Cancer immunotherapy mainly relies on the generation and stimulation of cytotoxic CD8 T lymphocytes (CTLs) within the tumor microenvironment (TME), priming T cells and establishing efficient and durable anti-tumor immunity. Therefore, there is a clear need to define and identify immunogenic T cell epitopes to use in therapeutic cancer vaccines. Naturally presented antigens in the human leucocyte antigen-1 (HLA-I) complex on the tumor surface are the main protagonists in evocating a specific anti-tumor CD8+ T cell response. However, the methodologies for their identification have been a major bottleneck for their reliable characterization. Consequently, the field of antigen discovery has yet to improve. The current review is intended to define what are today known as tumor antigens, with a main focus on CTL antigenic peptides. We also review the techniques developed and employed to date for antigen discovery, exploring both the direct elution of HLA-I peptides and the in silico prediction of epitopes. Finally, the last part of the review analyses the future challenges and direction of the antigen discovery field.

## 1. Introduction

The recent clinical success of antibodies targeting immune checkpoint molecules, such as programmed death receptor-1 (PD-1), its ligand PD-L1, and cytotoxic T cell-associated antigen 4 (CTL-A4), have led to a new and strong interest in the field of cancer immunotherapy [1,2]. Immune checkpoint inhibitors (ICIs) release the brakes of the immune system, reviving and boosting the effector function of specific anti-tumor T cells [3]. In 2018, James Patrick Allison and Tasuku Honjo received the Nobel prize for medicine “for their discovery of cancer therapy by inhibition of negative immune regulation” [4,5]. The overall response to ICIs is reportedly unsatisfactory for many types of cancer [6], highlighting the need to combine ICIs with cancer immunotherapeutic approaches, such as therapeutic cancer vaccines, with the artificial generation, stimulation, and tumor microenvironment (TME) infiltration of cancer-specific CD8+ T cells called cytotoxic T lymphocytes (CTLs) [2,7,8,9]. CTLs patrol the whole organism, checking the major histocompatibility complex (MHC), called the human leucocytes antigen (HLA) in humans, that possesses an antigenic peptide that is typically 8–11 aminoacidic residues in length and expressed on the cellular surface. If an aberrant peptide is eventually spotted in the HLA-I complex, the CTLs kill the cell.

In this context, to create effective tumor protection and rejection strategies, the reliable identification of tumor antigens in the HLA-I complex plays a pivotal role. To date, the methodologies for the correct identification of antigens in the HLA-I complex rely on the direct isolation of peptides from the HLA-I complex and/or the in silico prediction of relevant antigens [10,11].

The direct identification of peptides from the HLA complex is accomplished using different techniques, of which immunoaffinity purification (HLA immunoprecipitation) and the extraction of HLA peptides are the most well-established [12]. Isolated peptides are identified by tandem mass-spectrometry (MS/MS). The entire process is highly laborious and time-consuming [13,14]. It has been reported that immunoaffinity purification produces a low yield (i.e., 1–3%) [15], even though additional experiments in different settings are needed to better determine how much of the sample is lost [16]. The peptide isolation procedure is universally recognized as the bottleneck of the entire procedure and therefore requires improvement [16]. 

In the antigen discovery process, in silico tools are used to predict immunologically relevant antigens. The identification of target candidates relies on bioinformatic approaches that allow a structural analysis of genomic and proteomic data. For instance, next generation sequencing (NGS) approaches can benefit from the use of epitope prediction tools to combine two kinds of information: the differential gene expression in cancer compared to matched healthy tissue and the probability of those candidates to be presented on the cell surface onto the HLA molecule [17,18]. To achieve this, the actual bioinformatic tools contain T cell epitope algorithms that are able to predict putative T cell epitopes based on the well-characterized rules to which HLA-I presented peptides adhere [19,20]. Indeed, several predictors of HLA binders have been developed [21]; these will be defined in Section 4. HLA binding is only one part of the story; today, the entire processing machinery can be taken into account (e.g., proteasomal cleavage, transporter-associated antigen processing (TAP) transport) by the bioinformatic algorithms to predict relevant T cell epitopes [21]. 

NGS and in silico tools are effective methods in antigen discovery; however, improvements are needed. These methods lack rich and experimentally validated datasets, thus decreasing the accuracy of the predictive algorithms. The bioinformatic predictions give hints, but the selection of relevant epitopes from among the candidates always requires experimental validation [18,21].

This paper presents the information necessary to understand the antigen discovery field. It describes the concept of antigens, focusing mainly on CTL antigenic peptides. It then provides a concise analysis of the methods endorsed for the antigen discovery process, including in vitro and in silico approaches. It also highlights the gaps and challenges in the field. 

## 2. Antigens

The word “antigen” refers to the molecular structure seen by the antibodies or to any molecule or linear molecular fragment derived from the processing of the native antigen that can be recognized by T cell receptors (TCRs) [22]. This paper mainly examines CD8+ T cell-restricted antigens. The existence and role of tumor antigens have been discussed since 1940 and were reported on before the discovery of T cells, which occurred in the 1960s [23,24]. In 1943, Gross et al. performed a pivotal experiment that showed for the first time the role of the immune system in tumor rejection [25]. Briefly, mice were treated with methylcholanthrene and subsequently developed tumors. The tumors were then resected, and the tumor cells were implanted back into the mice. The mice then rejected the second tumor. This experiment demonstrated that acquired immunity was induced and directed against the tumor and that the rejection did not depend upon genetic differences between the inoculated mice and the mice that produced the tumor cells [25].

Later, Boon and colleagues achieved similar results using mutagen-treated murine cell lines that failed to form tumors in syngeneic mice; this research confirmed that tumors express antigens recognized by CTLs and confirmed the role of the immune system in rejecting malignant cells [26,27]. However, the molecular nature of the antigens expressed by tumors and recognized by CTLs was only discovered in 1989 by Lurquin et al. [28]. The researchers identified a single peptide recognized by CTLs that differed from the self-protein by single point mutation. This observation clearly showed that upon mutation, the tumors expressed altered proteins, thus labeling the cells for CTL recognition [28,29]. 

**Figure 1:**
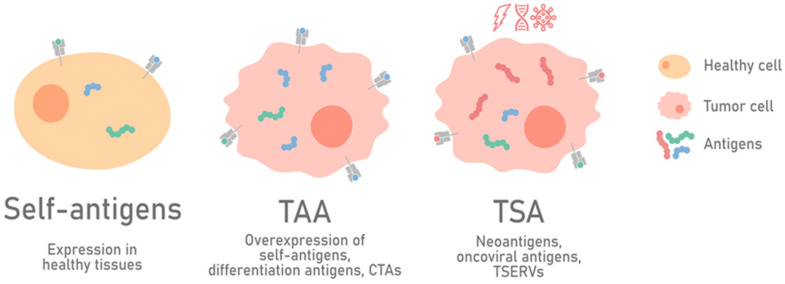
Classification of tumor antigens. Tumor antigens can generally be categorized into tumor-associated antigens (TAAs) and tumor-specific antigens (TSAs) based on the expression pattern of the parental gene. TAAs are self-proteins expressed in cancer cells; upon malignant transformation, the following consequences can be observed: the overexpression of normal proteins (gene overexpressed), the expression of proteins with tissue-specific gene patterns (differentiation antigens), or the expression of proteins derived from gene expression restricted to the testes (cancer germline/cancer testis antigens). TSAs are proteins expressed by tumor cells and can arise from mutations (neoantigens), from viruses involved in the oncogenic transformation (oncoviral antigens), or from the expression of tumor-specific endogenous retroviruses (*TSERVs*).

Today, based on the expression of the parental gene, tumor antigens can be classified into the general categories of tumor-associated antigens (TAAs) and tumor-specific antigens (TSAs) [30] (Figure 1).

### 2.1. Tumor-Associated Antigens

TAAs are a large family of antigens that includes antigens derived from genes overexpressed in tumors, differentiation antigens, and cancer germline/cancer testis antigens [30,31]. Antigens derived from genes overexpressed in tumors comprise a class of normal self-proteins which are minimally expressed by healthy tissues but constitutively overexpressed in cancer cells as a result of their malignant profile. The proteins which are usually overexpressed (e.g., EGFR, hTERT, p53, carbonic anhydrase IX) are mainly involved in the survival of the cancer cells and therefore are not susceptible to downregulation mechanisms, making them an attractive target for cancer therapeutic approaches [31]. For instance, in 1996, Gaugler et al. discovered an overexpressed antigen in renal cell carcinoma (RCC) called renal antigen 1 (RAGE-1) [32]. RAGE-1 is the first example of an antigen recognized by autologous CTLs in RCC, and its expression is restricted to the retina and to different histological tumor types. As the retina does not express HLA-I [33], RAGE-1 becomes a possible candidate to target for cancer immunotherapy [34,35]. Another interesting example is HER2/NEU, which is overexpressed in epithelial tumors such as ovarian and breast tumors [36,37]. The use of trastuzumab, a monoclonal antibody targeting the extracellular domain of HER2, has revolutionized the treatment of breast cancer HER2+ [38]. In addition, HER2 is a suitable candidate for the peptide vaccine approach; indeed, the nonapeptide E75, derived from the HER2 sequence, has been described as being able to elicit four different ovarian CTL cell line responses [39]. E75 as a monotherapy (Nelipepimut-S) or in combination with granulocyte macrophage colony-stimulating factor (GM-CSF) (NeuVAx) has been tested in clinical trials [40,41,42]. The genes belonging to the apoptosis pathway are also upregulated in tumor cells compared to healthy tissues, representing a source of T cell epitopes. For instance, a peptide derived from survivin, an apoptosis inhibitor protein, was used to generate CTLs in vitro from healthy donors; matched cell lines and primary malignant cells from patients were then lysed by those T cells [43]. In addition, a p53 derived wild type peptide (L9V) was used to generate in vitro CTLs that were able to kill squamous carcinoma cell lines in an L9V-HLA-dependent fashion [44]. 

As TAAs have a higher expression level in tumors compared to normal tissue and are shared among several tumors, a safe use for them has been proposed for cancer therapeutic approaches. For instance, novel chimeric antigen receptor (CAR) T cells have largely been adopted to target this class of antigens [31]. CAR T cells consist of an extracellular domain made of the variable region of the antibody’s heavy and light chains linked to the intracellular signaling domain (CD3-zeta, CD28, 41BB). This characteristic makes the CAR T cells able to kill the target in an HLA-independent manner [45]. The adoption of anti-CD19 CARs has showed good clinical outcomes in the treatment of B cell lymphomas and leukemias [46,47,48].

However, the main drawback in using TAAs in cancer immunotherapy is the feasibility of the expression analysis of these proteins in every single tissue under every physiological condition; this hinders a comprehensive safety profile of the TAAs [31]. Indeed, potential hazards (i.e., “on-target, off-tumor” toxicity, onset of autoimmune disease) associated with the clinical treatment based on these molecules have been reported. For instance, CAR T cells targeting carbonic anhydrase IX (CAIX) in RCC patients induced liver toxicity, requiring cessation of the treatment. Biopsies revealed CAIX expression in the bile duct epithelium with T cell infiltration, including CAR T cells; this is a typical example of “on-target, off-tumor” toxicity [49]. Moreover, the central and peripheral tolerance mechanisms eliminate the T and B cells’ ability to recognize self-antigens. A TAA peptide-based vaccine must break the tolerance, thus stimulating the low-affinity and rare T cells still circulating. This could interfere with the development of a proper cancer therapeutic vaccine [30,50]. The identification and use of an effective vaccine adjuvant could overcome the problem, delivering benefits to cancer patients [51].

The differentiation antigens represent normal proteins which are expressed as a consequence of a specific function of the target tissue. These were first reported in melanoma in which proteins (e.g., tyrosinase, Melan-A/MART-1, gp100/Pmel17) involved in melanin production or melanosome generation were often observed to be targets for CTLs from melanoma patients and healthy donors [35,52,53]. In the 1993 work of Brichard et al., lymphocytes derived from two melanoma patients were stimulated with irradiated cells from the autologous melanoma, and CTLs that were able to lyse them were obtained. The authors then demonstrated that the lysis was antigen HLA-0201-specific, and upon using the cloning approach, they identified the gene encoding the antigen as tyrosinase [53]. Rosenberg and colleagues administered tumor-infiltrating lymphocytes (TILs) to treat metastatic melanoma patients and had good results. The authors used the TILs to clone and determine the antigens involved in the anti-tumor response. In 1994, Rosenberg and colleagues established a CTL cell line called TIL1200 from a metastatic melanoma patient and were able to lyse the autologous melanoma and other melanoma HLA-0201+ cell lines. The authors showed that TIL1200 recognized a self-antigen not mutated in the HLA-0201 context and that the antigen sequence belonged to a membrane glycoprotein known as gp100/Pmel17 [54]. In the same year, Rosenberg and colleagues identified a shared and commonly expressed HLA-0201-restricted melanoma antigen that was recognized by T cells 1 (MART-1), applying a similar approach to that used to isolate gp100/Pmel17 [55]. In the following years, other melanoma differentiation antigens were identified, such as TRP1/gp75 and TRP2 (tyrosinase-related proteins), in the context of the HLA-A31 molecule [56,57]. Later, the TRP2 case was reviewed to identify an HLA-0201-restricted peptide, as HLA-0201 occurs frequently in the population. Based on the peptide-binding motif for HLA-A0201 and experimental validation, TRP2 peptides 180–188 were reported, and the sequence was found to be identical to the one recognized by H2Kb-restricted B16 murine melanoma CTLs, paving the way for a murine tumor immunotherapy model [58]. Furthermore, differentiation antigens such as prostatic acid phosphatase (PAP) and prostate-specific antigen (PSA) are used for prostate cancer immunotherapy. Naturally expressed HLA-02 restricted peptides from PAP were identified through sequence analysis and in vitro validation [59]. Moreover, two PSA HLA-02-restricted peptides which are capable of eliciting CTL responses have been reported by Correale et al. [60]. In addition, immunization experiments using HLA-0201 transgenic mice were employed to identify a peptide from carcinoembryonic antigens (CEA) [61]. This is a glycoprotein overexpressed in colon rectal cancer and other selected epithelial cancers, while in normal conditions, its expression is restricted to fetal development and in adults’ tissue in the epithelial cells of the gastrointestinal tract [62,63]. 

The main disadvantage in exploiting differentiation antigens as vaccines for cancer therapy is the onset of autoimmune toxicity. In cases of targeting melanoma-melanocyte antigens, reactions such as severe skin rashes and vitiligo lesions have been reported [64,65]. CAR T cells targeting CEA that were used to treat three patients with metastatic colorectal cancer showed a promising outcome in at least one patient; however, severe transient colitis was induced, most probably due to the CEA presence in the colonic mucosa [62]. These observations highlight the importance of targeting tumor-specific antigens and/or antigens with limited expression in normal tissues (e.g., cancer germline/cancer testis antigens).

Cancer germline/cancer testis antigens (CTAs) are a large family (the CT database consists of 204 genes [66]) of tumor-associated antigens expressed in human tumors of different histological origins but not in normal tissue, except for testis and placenta tissue [67]. In 1991, Van der Bruggen and co-workers identified the first human gene that encoded for a cancer testis antigen [68]. The authors isolated the CTLs that were capable of lysing the autologous cell line derived from the melanoma patient MZ2. Using a clonal sub-line and a DNA cloning approach, they identified the gene which was able to sensitize the sub-line to the CTL lysis. This gene was named melanoma antigen family A, 1 (MAGEA1), and it is expressed in many human tumors of different histological types. No expression was detected in normal tissues, with the exception of testis and placenta tissue [68,69]. In the same work, another two MAGE members were reported, MAGE A2 and MAGE A3 [68]. Subsequently, the nonapeptides derived from HLA-A1-restricted MAGE A1 and MAGE A3 were described [70,71]. 

After this, many antigens were identified in diverse tumors with restricted expression in testis tissue; Old and Chen called these cancer testis (CT) antigens [72]. To date, the MAGE family is subdivided into type I and type II. Type I comprises three sub-families, MAGE-A, -B, and -C, and contains the relevant CTAs. Type II includes the MAGE-D, -E, -F, -G, -H, and -L sub-families and Necdin that are expressed in different adult tissues [73,74,75]. Several members have been identified in the MAGE group [76], and MAGE A3 is one of the most frequently expressed TAAs in many tumors, including melanoma, non-small cell lung carcinoma (NSCLC), and head and neck tumors [77]. In melanoma, the preferentially expressed antigen of melanoma (PRAME) is another example of a CTA; PRAME is defined as a testis-selective rather than a testis-restricted CTA as its expression has been observed in endometrial, ovarian, and adrenal gland tissues in addition to testis tissue [78]. Building on the work by Ikeda and co-workers [79], Kessler et al. subsequently identified four HLA-0201 peptides that were restricted in PRAME [80]. In 2011, Quintarelli and colleagues were able to generate PRAME-specific CTLs from both healthy donors and leukemia patients [81]. In 1997, the serological analysis of recombinant tumor cDNA expression libraries (SEREX) technique was used by Chen et al. to identify novel tumor antigens in esophageal squamous cell carcinoma patients; the screening revealed sequences belonging to eight genes, among them New York esophageal squamous cell carcinoma 1 (NY-ESO 1), that were expressed in normal tissue, such as testis and ovarian tissue, and in several tumors, such as melanoma, breast cancer, bladder cancer, prostate cancer, and hepatocellular carcinoma [82]. Since then, NY-ESO1 has been employed in several immunotherapy-based treatments [83]. For instance, recombinant NY-ESO1 protein was used in combination with the adjuvant ISCOMATRIX in 46 patients with resected NY-ESO1+ tumors. Overlapping peptides were then employed to verify the T cells’ response to NY-ESO1 upon vaccination, showing CD4+ and CD8+ T cells’ specific response to both known and uncharacterized peptides derived from NY-ESO1 [84]. In 1997, Türeci et al. used the SEREX technique to investigate human melanoma and described a novel CTA gene called synovial sarcoma X chromosome breakpoint (SSX2) [85], to which a T cell response has been reported in patients with tumors of diverse histological origins [86]. An analysis of the sub-clones derived from the MZ2 melanoma patient allowed the identification of the B melanoma antigen (BAGE) gene and the HLA-Cw1601-restricted peptide [87]; BAGE expression was found only in testis and tumor (e.g., bladder cancer) tissues, and thus was found to be a member of the CTA family. Using a similar experimental procedure, other CTAs were identified—the gene G antigen 1 (GAGE) and the HLACw0601-restricted peptide [88].

Being immunologically privileged sites, the testes and placenta do not express HLA molecules [89]; therefore, CTAs are promising targets for cancer immunotherapy. Indeed, MAGE A3 as a recombinant protein has been used in the largest ever phase III lung cancer clinical trial, the MAGE A3 as Adjuvant Non-Small Cell Lung Cancer Immunotherapy (MAGRIT) trial [90]. In phase II, the vaccination protocol was well tolerated and the results promising; however, MAGRIT failed to show any improvement in disease-free survival (DFS) when compared with a placebo [91]. In addition, TCR gene modified T cells to target the MAGEA3/A12 HLA-A0201 restricted peptide were used to treat nine patients with tumors expressing MAGE A3/A12 in a phase I/II clinical trial. The encouraging cancer regression that was observed was dampened by severe neurotoxicity that resulted in the deaths of two patients. Subsequent analysis revealed the expression of MAGE A12 in the brain, explaining the inflammation and neuronal degeneration that was observed [92]. The use of CTAs, such as MAGE family members, is promising; however, caution is required in further applications. 

### 2.2. Tumor-Specific Antigens

TSAs are a category of antigens restricted to tumors and are not found in healthy cells; this is the result of malignant mutations or the expression of viral elements. Neoantigens, oncoviral antigens, and endogenous retroviral elements belong to this category [30,35,93].

Neoantigens are a subset of TSAs produced as a direct consequence of genetic alteration caused by tumor DNA mutations (e.g., non-synonymous single point mutations, frameshifts, insertions/deletions) and are patient-specific [94,95]. In 1995, Coulin et al. reported the first example of a neoantigen [96]. The authors identified the source gene of the antigen that was recognized by an autologous CTL and called it melanoma ubiquitous mutated (MUM-1). As the gene was expressed ubiquitously, the authors wondered whether a mutation had occurred in the gene to induce an anti-tumor T cell response. Actually, MUM-1 in the melanoma cells carried a single point mutation that resulted in an amino acid change in the nonapeptide HLA-B44 restricted, making it suitable for interaction with the T cells [96]. Several other neoantigens have since been identified. For instance, Rosenberg and colleagues discovered a single point mutation in the β catenin sequence in one melanoma patient that resulted in a change from a serine to a phenylalanine residue at position 37; the derived peptide (SYLDSGIHF) showed a high affinity binding for HLA-A24, the patient’s HLA allele [97]. Moreover, a single point mutation in the gene encoding CDK4 resulted in the generation of an HLA-A0201-restricted peptide that was recognized by autologous CTLs in the melanoma patient, and the mutation altered the cell cycle regulation [98]. A mutation in the CASP-8 gene that reduced the function of the protein and generated an HLA-B3503-restricted peptide that was recognized by autologous CTLs was reported in a patient with squamous cell carcinoma of the oral cavity [99]. 

The neoantigens in cancer immunotherapy have the undiscussed advantage of being non self-antigens and hence the T cells are not affected by central tolerance, making them highly immunogenic antigens [94]. However, their main limitation is their great variability within and between tumors. Great variability within tumors can induce negative selection, allowing the survival of the cancer cells that no longer express the neoantigens. Great variability between tumors requires characterization and the development of a specific vaccine for each patient. In addition, the mutational burden plays a pivotal role in the generation of neoepitopes. Tumors with a high mutation frequency are most likely to generate neoantigens. Tumors with a low mutation burden may be difficult to investigate in terms of identifying neoantigens and subsequent vaccine production [31,35]. 

Oncoviral antigens consist of proteins derived from the viruses driving the oncogenic transformation; these proteins are the source of peptides present on the cellular surface in the HLA context and recognized by T cells. As oncogenic viruses are shared by the same kinds of tumors, this class of antigen is not patient-specific [30,35]. Prophylactic vaccines have been produced and mainly rely on eliciting neutralizing antibodies to prevent the virus from entering the cells. The treatment of established tumors involves targeting T cell epitopes [30]. For instance, human papillomavirus (HPV) is associated with benign papilloma or warts and cancer of the cervix, anus, penis, and head and neck [100]. Ramos et al. isolated PBMCs from patients with HPV+ cancer and stimulated them in vitro with a mix of HLA-I-restricted and HLA-II restricted peptides, covering the E6/E7 proteins of HPV16; the authors were able to demonstrate that the patients had E6- and E7-specific T cells [101]. These have been called HPV-ST, and an on-going phase I clinical trial is evaluating the effect of HPV-ST cells in treating HPV+ tumors (NCT02379520). Another example is the development of therapeutic vaccines for cancers related to the Epstein–Barr virus (EBV) which is involved in the onset of several disorders, such as B-cell lymphoproliferative disorders and nasopharyngeal carcinoma [102,103]. In a phase I clinical trial, 16 patients with established HBV+ tumors were treated with modified vaccinia Ankara (MVA) encoding the full length of LMP2 and the C-terminal of EBNA1 proteins from EBV. A specific T cell response to LMP2 and/or EBNA1 was detected, showing the feasibility of boosting an EBV-specific immune response [104]. However, the extent of the clinical benefits is still being investigated in a phase II clinical trial (NCT01094405). Oncoviral antigens lack expression in healthy cells, making them highly tumor-specific. They are also common to patients. However, 15% of cancers have a viral etiopathology, limiting their clinical application [105,106].

Endogenous retroviral elements (ERVs) or human endogenous retroviral elements (HERVs) are fragments of genomic DNA derived from the integration of retrotranscribed retroviral RNAs that infected the germ line cells of humans’ ancestors. Over time, ERVs have been vertically transmitted and, to date, they represent 8% of the human genome. ERVs have accumulated mutations over time, losing the capability of producing competent replicative viral particles [107]. Moreover, epigenetic mechanisms (e.g., methylation) suppress most of their expression in healthy cells. For instance, in the thymus, ERVs are partially epigenetically silenced, and ERVs which are reactive to T cells do not go through complete negative selection [108]. In cancer, ERVs are induced upon malignant transformation and/or epigenetic therapy, becoming targets for cancer therapeutic approaches [109]. In 2015, Rooney at al. investigated the cytolytic activity and expression pattern associated with 66 ERVs in tumors compared to healthy tissues. Surprisingly, they found three ERVs (ERVH5, ERVH48-1, and ERVE4) with minimal or undetectable expression in normal tissue and overexpression in tumors; these have been termed tumor-specific endogenous retroviruses (TSERVs) [93]. Interestingly, in regard to ERVE4, Rooney´s data had already been experimentally validated. For instance, following hematopoietic stem cell transplantation (HSCT), RCC patients experienced disease regression. Child and colleagues found RCC-reactive CD8+T cells derived from the donor. Using a cDNA cloning approach, the authors identified the antigen in HERV-E gene products and described an HLA-A11-restricted 10-mer peptide (ATFLGSLTWK) as the target recognized by the tumor-reactive CTLs [110]. The same authors also reported the identification of three HLA-A0201-restricted peptides derived from HERV4*env* that were able to elicit RCC-reactive CTL responses [111]. ERVH-5 has been reported in bladder, colorectal, head and neck, lung squamous, ovarian, stomach, and uterine cancers. ERVH48-1 is prominently expressed in bladder cancer and prostate cancer [109]. Schiavetti et al. described CTLs which were reactive against the peptide derived from HERV-K-MEL in two melanoma patients. The authors determined that the peptide sequence (MLAVISCAV) was HLA-A2-restricted, showing that the CTLs’ reactivity against the peptide occurred only in the two patients and not in the healthy donors [112]. Based on the presence of HERV-K gag proteins in the cytoplasm of primary tumor cells and on the detection of antibodies to HERV-K gag in patients with seminoma, Rakoff–Nahoum investigated the HERV-K-specific T cell-mediated immune response in the blood of those patients. The authors synthetized 15 HERV-K predicted peptides based on the HLA-I binding motif and proline-enriched region. Next, PBMCs from seminoma patients and healthy donors were screened with four pools of these peptides. The T cell reactivity was higher in at least three pools of peptides in the seminoma patients compared to the healthy donors [113].

Their high tumor specificity and expression [93] and incomplete T cell tolerance [108] make ERVs the ideal target for cancer immunotherapeutic approaches. In addition, autologous CTLs which are able to recognize HLA-restricted peptides have been reported [110,111,112,113], and ERVs are common to cancer patients. This allows for off-the-shelf therapy. However, the epitopes recognized by CTLs which are found in cancer patients are still few, and the expression of different HERV families in cancer is still limited. Therefore, future proteomic analysis, especially of the thymus, and an in-depth understanding of the mechanisms involved in HLA-I presentation will shed light on the use of ERVs in cancer immunotherapy [109].

## 3. In Vitro Methods for HLA Ligand Enrichment

### 3.1. Immunopeptidome

**Figure 2:**
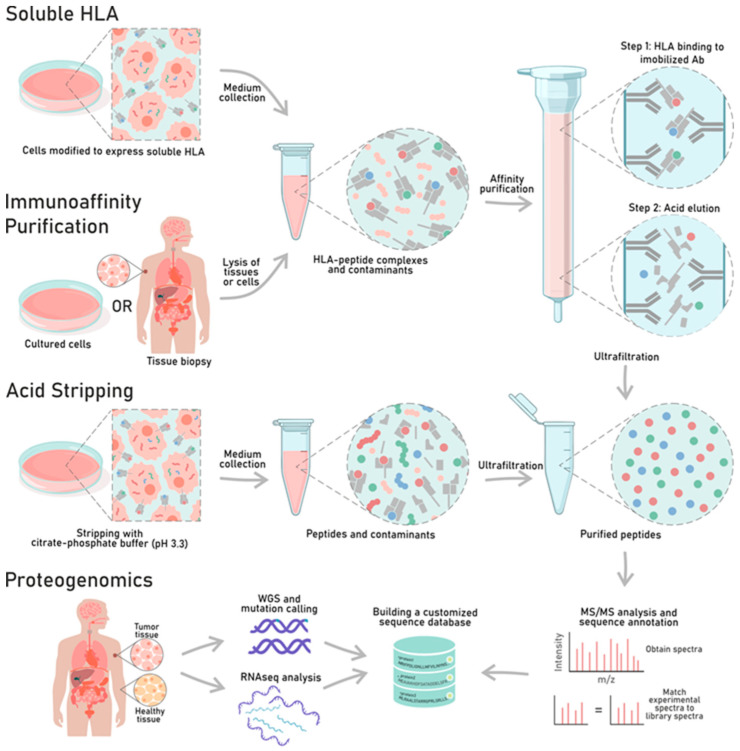
Workflow of the main approaches for the isolation of human leucocytes antigen (HLA)-I restricted peptides. In vitro methods for HLA enrichment are depicted. The direct isolation and identification of the peptides present in the HLA complex can be achieved through different approaches, such as the use of acid stripping, soluble HLA, immunoaffinity purification, and the proteogenomic. The purified peptides are then resolved by mass spectrometry analysis, and the spectra can be searched against a conventional database or a customized database (proteogenomic approach).

The existence and role of tumor antigens in eliciting a specific anti-cancer immune response, combined with the discovery of CD8+ T cell sub-populations which are able to recognize and kill tumor cells in an HLA I antigen- restricted manner, make the identification of epitopes recognized by CD8+ T cells a priority in the cancer therapeutic field. The peptides which are bound to the HLA complex and found on the cellular surface are referred as immunopeptidomes or ligandomes. The methods developed to study and analyze these are known as immunopeptidomics. The aim of immunopeptidomics is to reliably identify immunopeptidomes and thus guide the development of cancer therapeutic vaccines. The direct isolation of HLA peptides from the cell surfaces can be accomplished using different techniques. In this section, we describe the past and present approaches to the direct isolation of peptides in order to investigate the immunopeptidome landscape, highlighting the advantages and disadvantages of each (Figure 2).

#### 3.1.1. Acid Stripping 

In 1993, Storkus et al. published for the first time a method for the direct isolation of peptides from the HLA-I complex based on acid stripping. The method consisted of treating the cells with a citrate–phosphate buffer at pH 3.3 for a period as short as 15 s, thus allowing the cells to remain viable and become phenotypically class I-deficient [114]. After the treatment, a flow cytometry analysis of the acid-treated cells revealed the retention of class I heavy chains of the HLA complex in the cell membrane, while the class I light chain (β-2 microglobulin) was absent. Since β-2 microglobulin is essential to stabilize the binding of the peptide within the complex, its dissociation is directly associated with the release of HLA-bound peptides. Storkus et al. collected the supernatant from acid-treated, influenza A strain-infected cells and separated the fractions using reverse-phase high performance liquid chromatography (RP-HPLC). Next, the fractions were analyzed for their capacity to sensitize a B cell line to lysis which was mediated by a CTL line that was specific to the influenza A matrix peptide (Flu M1 57-68). Thus, the fractions that were able to induce the lysis were identified. The fractions contained a peptide with a sequence similar to the Flu M1 58-66 sequence [114]. 

Following this work, the same authors used the aforementioned method to extract peptides from human melanoma cell lines. The subsequent analysis revealed six peptides (P1–P6) that were recognized by HLA-A2-restricted TILs [115]. Subsequently, the method has been used to investigate HLA-bound peptides in melanoma [116] and to isolate the first leukemia-specific immunogenic peptide derived from the Bcr-Abl fusion protein [117] in order to develop peptide-based immunotherapy.

Acid stripping can be used to extract peptides from adherent and suspension cell lines. Moreover, the approach is quite simple and cost effective. However, acid-treated cells are often damaged in the process, releasing proteases that generate peptides from either abundant cell protein or from cytoplasmic protein. Consequently, the peptides are often contaminated with non-HLA-restricted peptides [11,118].

#### 3.1.2. Soluble HLA Molecules

The use of soluble HLA molecules is based on cells engineered with the vector encoding the desired soluble HLA complex. This approach requires the generation and isolation of stable transfected cells to express the soluble HLA molecule. The latter lacks the transmembrane domain that is needed for it to be secreted. Hence, the soluble HLA is released in the cellular medium that is collected and immunopurified for the further characterization of peptides [11,119]. 

Different approaches for generating the soluble HLA-I complex have been used over the years. In 1986, McCluskey and co-workers developed a soluble HLA molecule by fusing the non-functional transmembrane carboxyl terminal C2 domain of Q10^b^ with the polymorphic amino-terminal N and C1 domains of H-2D^d^, thus generating a chimeric H2D^D^/Q10^b^ molecule [120]. In the following years, another group realized a soluble HLA as a fusion protein between the extracellular domain of H2Kb and the immunoglobulin heavy chain polypeptide [121]. A third approach to generating soluble HLA has been reported by Grumet et al. The authors removed the transmembrane and cytoplasmatic regions from the B7 gene, creating soluble HLA-B07 (sHLA-B7) that in in vivo experiments suppressed humoral alloimmunization [122].

Soluble HLA molecules have been extensively used in investigating the immunopeptidome landscape in order to identify novel peptide candidates for therapeutic vaccines in the fields of cancer immunotherapy [123,124] and infectious diseases [125,126,127,128]. The use of soluble HLA for peptide isolation has been proposed as a valid alternative to immunoaffinity purification [119]. In some cases, the yield of retrieved peptides has been reported as improved compared to immunoaffinity purification [124]. Nevertheless, the approach is not feasible to study patient tumor samples or tissue because it requires the generation of stable transfected cells.

#### 3.1.3. Immunoaffinity Purification

Immunoaffinity purification relies on the direct isolation of HLA-I complexes from solubilized samples that are subsequently applied to columns and previously coupled with monoclonal antibodies that are able to bind the desired HLA-complex. The peptides are isolated from the HLA complex through acid elution and purification. The sequence is resolved by tandem mass spectrometry (MS/MS) analysis and validated in in vitro and in silico assays (e.g., motif clustering analysis) [11].

Briefly, the protocol starts with selecting the material to process; this material can be cells, tissue, biopsies, or biological liquid (i.e., plasma) [129]. Cells are the simplest material to work with because it is possible to expand them in culture and preserve the pellets by freezing them at −80 °C for up to 6 months [11]. The amount is usually in the range of 5 × 10^7^ and 1 × 10^9^ cells [11]. Next, the material is lysed using a lysis buffer that is a combination of a 0.5–2% solubilization reagent (e.g., Igepal, sodium deoxycholate, CHAPS), salts, and protease inhibitors [11,14,118]. The cell lysate is then centrifugated and the supernatant applied to the columns coupled with a monoclonal antibody. 

The choice of monoclonal antibodies varies depending on the experimental conditions according to the investigated material and to the desired HLA complex. After the HLA complex binding, the columns are washed extremely well to get rid of material that is not specifically bound, detergent that could interfere with the downstream mass spectrometry analysis, and salts that could form crystal precipitates. The final step is the elution of the peptides from the HLA complexes. This is achieved using an acid solution with a pH of 3.0 (e.g., 10% acetic acid). Purification of the peptides is carried out using C18 columns or low-protein binding with a molecular weight cut-off (MWCO) spin filter. The fraction containing the HLA peptides is then vacuum-dried to reduce the acetonitrile concentration and analyzed by mass spectrometry to resolve the amino acid sequences [11,14].

An early attempt to isolate HLA-I antigens using immunoaffinity purification was reported by Parham in the 1970s [130]. In this work, the author demonstrated the feasibility of isolating the HLA-A02 and HLA-B07 complexes’ antigens, starting from the crude membrane of the JY cell line. The sample was applied to a series of columns, each one respectively coupled with the monoclonal antibodies anti-HLA-A02, anti-HLA-B07, and anti-pan HLA-I. This first attempt paved the way to studying the ligandome landscape using immunoaffinity purification. Immunoaffinity purification evolved profoundly over the subsequent years and has been used to systemically analyze the HLA-restricted peptides derived either from virus- or cancer-transformed cells, as shown in the work of Engelhard and Hunt [131,132,133,134,135,136], Natheson [137], and Rammensee [138]. For instance, immunoaffinity purification has been used to identify the peptides that are recognized by CTLs derived from patients, as shown by Cox et al. Cox et al. isolated peptides from melanoma cells via immunoaffinity purification; nine peptides were found to be recognized by CTLs derived from different patients, showing the feasibility of identifying candidates for peptide-based vaccines [132]. Immunoaffinity purification has been used to investigate antigens in malignant and non-malignant samples in order to define a pool of tumor-restricted peptides. This approach led to the successful identification of the peptides which are exclusive to tumors that are able to activate pre-existing T cells in colorectal carcinoma patients [138]. Interestingly, immunoaffinity purification can be used to identify spontaneous T cell responses in malignancies with few described associated antigens; for example, in chronic lymphocytic leukemia [139].

Immunoaffinity purification results in highly specific HLA peptides; however, the number and quality of the retrieved sequences is strongly affected by the following circumstances. Firstly, the material to be processed has to be provided in large amounts (5 × 10^7^–1 × 10^9^ cells), preventing the analysis of small and often clinically relevant tissues (e.g., needle biopsies) [11,140]. Secondly, the expression level of the HLA varies according to the samples, thus impacting the final number of peptides. Moreover, specific anti-HLA antibodies are not always available or are not optimal for binding. Finally, the use of spin filters has been associated with polymer contamination and peptide loss [11].

### 3.2. Proteogenomics

Proteogenomics is a broad research area that combines knowledge from the proteomic and genomic fields. Indeed, it combines the rapidly developing MS/MS approaches in proteomics with the high-throughput sequencing methodologies of genomics. The aim of proteomics is to identify and characterize (e.g., cellular localization, signaling pathway) the proteome of a given species. From a technical point of view, this aim is achieved through the digestion of proteins to generate peptides. Regarding the analysis of the immunopeptidome landscape, peptides are isolated from HLA-I complexes according to one of the aforementioned methods (Section 3.1.1, Section 3.1.2, and Section 3.1.3). The peptides are then resolved by MS/MS analysis, and the generated spectra are searched against the theoretical spectra of all candidate peptides, as represented in conventional reference databases (e.g., EntrezProtein, UniProtKB). These databases contain all the protein-coding sequences in the genome. Mutations, products of novel coding proteins or of annotated non-coding regions, and frameshifts cannot be identified because the spectra do not match any references in the canonical database [141], hindering the identification of potential vaccine peptides such as neo-antigens. 

Genomics examines the genome organization (e.g., gene–gene interactions) within a given organism and mainly relies on sequencing approaches (whole genome sequencing, RNA sequencing) and bioinformatic algorithms for the subsequent interpretation of data. The introduction of next-generation sequencing (NGS) and the advancement of bioinformatics have revolutionized our approach to DNA and RNA sequence analysis, paving the way for tailored therapeutic cancer treatment. With respect to HLA-restricted tumor antigens, sequencing methods direct the identification of patient-specific neoantigens in therapeutic settings, taking advantage of whole exome sequencing-based mutation calling [94,142]. In 2017, Sahin et al. reported the first example of neo-epitope prediction, which was applied to the development of a cancer vaccine for the treatment of 13 melanoma patients [143]. The authors identified nonsynonymous mutations by the comparative exome and RNA sequencing (RNA-seq) of tumor biopsies and healthy blood cells. They then prioritized the neo-epitopes with predicted high affinity binding to the autologous HLA and a high expression level of RNA. This led the authors to develop an RNA vaccine encoding poly-neoepitopes for personalized melanoma treatment [143]. Following a similar approach, Wu et al. created a vaccine to target personal neoantigens. The authors used a whole exome sequencing of a patient’s tumor and normal cells to identify somatic mutations and RNA sequencing in order to confirm the mutation expression. Peptide selection was based on the predicted binding affinity to the autologous HLA, developing synthetic long peptides containing up to 20 neoantigens per patient [144]. Even though knowledge of the genome and/or the RNA expression level allows researchers to make inferences about the use of HLA-I peptides for a therapeutic cancer vaccine, the genomic approach by its very nature does not take into account post-translational modifications such as methylation, phosphorylation, and glycosylation. These have reportedly been involved in eliciting T cell immune responses and can be identified solely using MS/MS approaches [145,146,147]. For instance, phosphorylated peptides, but not their unphosphorylated homologous peptides, were recognized by CD8+T cells [146]. In addition, sequencing analysis lacks authentic knowledge of the HLA presentation, as the complex pathway underlying the presentation machinery cannot be completely deduced from the sequencing methodology and downstream bioinformatic analysis.

In 2004, Church et al. for the first time combined the potential of proteomics with global genome annotation, developing a new method for mapping the peptides detected in *Mycoplasma pneumoniae.* The method was named “proteogenomic mapping” [148]. Thus, proteogenomics as a science was born in 2004 in order to integrate genome annotation and proteomic application [141].

Applied to HLA ligandome analysis, proteogenomics addresses two main issues—the actual presentation of HLA peptides in the genomic analysis and the need for patient- or sample-specific mutation databases in order to search for spectra data [149]. The main rationale is the use of exome sequencing mutation calling in order to identify mutations in tumor samples and RNA-seq to confirm their expression. The sequencing data are then used to assemble tailored databases to investigate the proteomic data in the form of MS/MS spectra which are derived from the immunopeptidome analysis of the same samples. This approach has been successfully applied to the identification of HLA-restricted tumor neoantigens [150,151,152].

Recently, Bassani–Sternberg and colleagues described a proteogenomic pipeline for the identification of non-canonical peptides (long non-coding genes, UTRs, transposable elements, pseudogenes) from the ligandome repertoire [153]. Using MS/MS, the authors compared the immunopeptidome landscape of seven patient-derived melanoma cell lines and two pairs of lung cancer samples with matched healthy tissues. Using the same sample, they performed exome sequencing mutation calling and RNA sequencing analysis. The MS data were then compared against a personalized database built from the translation of the transcripts acquired from the sequencing analysis. This approach identified hundreds of non-canonical HLA peptides [153]. The combination of exome sequencing and MS/MS was also used in Kalaora et al. to investigate immunogenic neoepitopes in human melanoma [154].

Proteogenomics is a promising method to shed light on the antigen landscape, especially to identify neoantigens which are otherwise not detectable with conventional approaches. However, the amount of samples required for proteomic analysis is still high, hampering the investigation of small amounts of material (e.g., needle biopsies) [155].

## 4. Prediction of T Cell Epitopes

**Figure 3:**
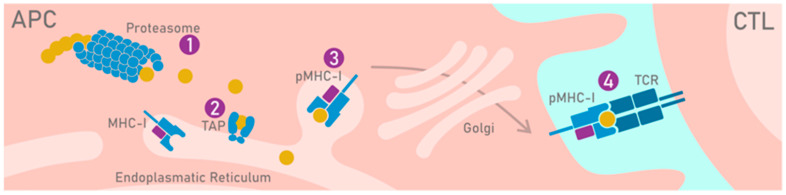
Processing and presentation pathway of HLA-I-restricted peptides. Intracellular proteins are processed by a proteasome (1). This cleavage determines the C-terminus of the peptides. The peptides bind transporter-associated antigen processing (TAP) that transports them into the endoplasmic reticulum (ER) (2). The peptides bind the HLA molecule that forms the pHLA complex (3) that is transported via the Golgi on the cell surface where CD8+ T cells can recognize them (4).

As mentioned, CTLs control the health of an organism, recognizing linear peptides which are presented on the MHC or, in humans, the HLA molecule. This system allows T cells to scan intracellularly processed proteins and consequently have an indication of the metabolic state of the cells; for example, whether they are transformed (cancerous) or virally infected. Protein degradation is mainly performed by the proteasome; however, the contribution of other proteases at this point seems clear [156]. The cleaved peptides are then transported in the endoplasmic reticulum (ER) by an ATP-binding complex called transporter-associated antigen processing (TAP). Other mechanisms of peptide translocation into the ER are evident yet poorly understood [157]. In the ER, peptides bind to the MHC molecule and are transported to the cell’s surface, where the complex (pMHC) will be potentially recognized by the TCRs of CD8+ T cells (Figure 3).

### 4.1. Prediction of Antigen Processing and Presentation

#### 4.1.1. Proteasomal Cleavage Prediction

The proteasome is a multi-catalytic protease complex that is formed by different sub-units and represents the cellular main center of the degradation of misfolded or un-needed proteins. The core of the proteolytic machinery is the 20S complex that contains three catalytic sub-units (β1, β2, and β5) that have specific catalytic activity: peptidyl-glutamyl-peptide-hydrolyzing activity (cleavage after acidic amino acids), trypsin-like activity (cleavage after basic amino acids), and chymotrypsin-like activity (cleavage after large, hydrophobic amino acids), respectively [158]. In the context of antigen presentation, proteasomes have been experimentally shown to be involved in the production of the C-termini of peptides [159,160].

Cells which are exposed to IFNγ substitute the constitutive β1, β2, and β5 with the homologs LMP2 (β1i), MECL1 (β2i), and LMP7 (β5i) that are encoded in the same locus as the HLA molecules and for this reason are historically associated with “immune”-related activity. This newly formed protease complex is called immunoproteasome, and its catalytic activity is characterized by reduced cleavage after acidic amino acid residues and increased cleavage after hydrophobic and basic residues [158]. Proteasomal cleavage is the first step in the antigen processing machinery; therefore, significant effort has been made to develop models that are able to predict its activity.

Currently, the tool which is generally recognized as the best performing tool to predict both proteasomal and immuno-proteasomal cleavage is NetChop 3.1 [161]. This is an updated version of a previously developed artificial neural network (ANN) architecture [162]. Benchmarked with other proteasome cleavage prediction tools, NetChop has been shown to be better at predicting MHCI epitopes. It has been speculated that NetChop is better at predicting naturally processed epitopes because it is trained with HLA ligand data, unlike other tools that have been trained with in vitro proteasomal cleavage data only. This might have allowed the model to better generalize the different contributions coming from additional proteases [156]. Interestingly, proteasomes can also generate different kinds of epitopes by joining non-contiguous regions through a process called post-translational peptide splicing [163,164]. However, bioinformatic tools which are able to predict this phenomenon are currently lacking.

#### 4.1.2. TAP Binding Prediction

The TAP complex is a heterodimer composed of two proteins, TAP1 and TAP2. It is an ATP-binding transporter delegated to the transport of peptides from the cytosol into the ER. Here, the peptides might be further processed by aminopeptidases or directly loaded onto the MHC and successively transported through the cell’s surface. It has been shown that the TAP transporter prioritizes peptides of certain lengths and carboxyl terminus residues commonly found to be HLA class I anchors, with minor contributions (positive and negative) from residues in other positions. It has therefore been speculated that there is a coevolution of TAP with the MHC (despite its high polymorphism) [158]. Models to predict TAP’s transport efficiency with peptides of arbitrary length have been produced over time [165,166], showing some improvement in epitope prediction efficiency. This approach has never been very successful alone; however, it has found a discrete use in combination with methods that will be discussed later.

Recently, it has been demonstrated that TAP-independent transport has a relevant role in CTL epitope production [157,167]. However, the possible mechanisms and the weight that it has globally on epitope presentation remain largely unknown [168].

#### 4.1.3. Peptide–MHC Binding Prediction

HLA is considered the most polymorphic protein of the human genome (hyper-polymorphic protein). The class I region, located on chromosome 6, encodes for genes that form HLA class I molecules. Interestingly, just the HLA-A, -B, and -C gene polymorphisms account for the almost 17,000 different alleles which have been annotated so far [169]. Notably, despite the incredibly large number of alleles, the HLA class I molecules can be clustered into nine groups called “supertypes” that reflect close peptide-binding specificity [170]. However, each allele in a supertype has a unique and specific peptide-binding preference. Therefore, the fact that a peptide can bind to a given allele does not necessarily indicate that it can also bind to other alleles of the same supertype [171].

##### HLA Motif Deconvolution

Despite the constant increase in MS-eluted immune peptidome data [172,173,174], HLA peptide deconvolution—the process of associating each ligand to its presenting HLA molecule(s)—is still a critical task. The tool most commonly used to this end is GibbsCluster 2.0. It performs sequential unsupervised alignments and clustering tasks of peptide sequencing based on Gibbs sampling. The latest version can also handle variations in peptide length for the generation of a motif [175,176,177].

Interestingly, it has been shown that incorporating deconvoluted HLA peptidomics data can improve the accuracy of HLA-binding prediction tools for those HLA alleles with still few ligands in the existing databases [178]. This approach can be used to refine our understanding of peptide–HLA interaction when considering peptides which are longer than nine amino acids (generally the most common and studied length), as the motif can change slightly when longer ligands interact with HLAs [178]. This technique has been shown to help increase the yield of immunopeptidome runs by 20–30% [179].

##### MHC-Binding Affinity Prediction

MHC alleles may differ because of different amino acid substitutions, most of which are found within the binding site and are critical to determining peptide motif recognition [170]. One of the most crucial steps in the antigen presentation pathway is the ability of the peptide to form a complex with the HLA molecule, thus determining its probability of being presented. The ability to predict the peptide affinity for the HLA is generally recognized as a key factor in the selection of T cell epitopes. The first attempts to predict peptides’ MHC-binding affinity relied on motif search techniques, but today, machine learning-based methods are preferred. 

One of the most commonly used tools to predict MHC-binding affinity is NetMHC 4.0 [180], a feed-forward ANN ensemble with a single hidden layer which has been trained with a set of quantitative peptide-MHC class I affinity measurements from the Immune Epitope Database (IEDB) of peptides of different lengths. Another ANN-based tool is MHCflurry [181]. This is an open source package, and its architecture combines the use of locally connected and fully connected layers which have been trained with both experimental affinity measurements and MS-eluted peptides for allele-specific prediction. 

Other methods are called pan-specific as they accept both peptide and HLA amino acid sequences and are able predict the affinity of any peptide to any HLA-I.

NetMHCpan is the most commonly used tool [182], and in its latest version (NetMHCpan 4.0) [183], an increased prediction accuracy has been reached by adding MS-eluted peptide data into the training set.

Interestingly, PSSMHCpan [184] is a pan-specific HLA-binding affinity method based on allele-specific position-specific scoring matrices (PSSM) which are generated by multiple sequence alignments of peptides binding to already characterized HLAs. When uncharacterized HLAs are encountered, the algorithm uses the similarity to the nearest HLA sequence as a weight that affects the binding score for the queried HLA.

In recent years, deep learning, a particular neural network architecture, has shown its power in classification and regression tasks and has attracted increasing attention. Zhao et al. [185] developed a convolutional neural network that uses different peptide properties, such as the order of the sequence, the hydropathy index, polarity, and the length of peptide needed to perform the prediction, as these are the key factors in determining the binding to the HLA molecule. Zeng and Gifford developed a deep learning-based method that consists of a binding affinity prediction module and a peptide embedding module [186]. The latter applies a deep language model to embed each peptide into a vector representation. The concatenated output of the two models represents the input for a fully connected neural network (NN). Zeng and Gifford also suggested a residual neural network (PUFFIN) that quantifies the uncertainty in peptide–HLA affinity prediction or, in other words, determines the confidence of the prediction [187]. Another recently developed pan-specific method is ACME [188], which consists of a deep convolutional neural network (CNN) implemented with an attention mechanism that returns interpretable patterns that facilitate the prediction. DeepHLApan is a recurrent neural network (RNN)-based method that takes neoepitopes as inputs and considers both the possibility of mutant peptide presentation and its potential immunogenicity [189]. 

Over time, sequence-based machine learning-driven methodologies have become increasingly popular because of their predictive power. However, they give poor structural insights into the dynamics of the interactions between the peptides and the HLA molecules. Modeling approaches are suitable tools to tackle this problem, but their use has been hindered for many years because of the high computational power required. In recent years, different tools have been developed for this purpose, or existing tools have been implemented to enhance their performance. One such tool is the web-server tool DockTope [190], currently hosted by IEDB (http://tools.iedb.org/docktope/). DockTope is able to iteratively model pMHC-I complexes until the best conformation is found, but given the small number of crystal structures of pMHC complexes that is available, the tool is only able to model the conformations of two human (HLA-A*02:01 and HLA-B*27:05) and two murine (H-2-Db and H-2-Kb) MHC-I allotypes. DINC [191] is another web-server tool based on the incremental meta-docking approach that has high accuracy in predicting the geometry of peptides in a complex with MHC molecules. GradDock [192] is a fast and accurate structure modeling algorithm for pMHC-I. The docking simulator was designed to be unbiased toward any MHC-I molecules by generating the initial unbound peptides in vacuo and successively inserting them into the desired MHC-I molecules. Finally, APE-Gen [193] is a very fast and accurate modeling method that has the potential to be useful because of its scalability (i.e., modeling thousands of pMHCs or non-canonical longer peptides) and flexibility.

#### 4.1.4. Combination of Different Predicting Tools

In order to better mimic all the processing and presentation steps that a naturally presented epitope goes through, approaches that combine different single step predictors have been produced. Tools such as NetCTL and NetCTLpan have shown that the combination strategy is more effective than individual tools in predicting naturally presented epitopes [194,195]. NetMHCcons combines the predictive power of three state-of-the-art peptide–MHC binding predictors and has been shown to outperform individual tools [196]. The ultimate advantage of using a combination of the previously described methods is that the number of candidate peptides to be tested is significantly reduced compared to predictions based on individual methods. Nevertheless, a comprehensive benchmarking of all these tools has never been performed.

### 4.2. Prediction of Epitope Immunogenicity

At this point, it is important to note that the ability of a peptide to bind the HLA molecule and be present on the cell’s surface does not imply its ability to engage a T cell and promote its activation. There is a difference between naturally presented epitopes; some are able to elicit an immune response (and, hence, are immunogenic) and some are not.

#### 4.2.1. Peptide Stability Prediction

A peptide’s affinity to a specific HLA molecule is crucial for its presentation, yet it does not give any indication about its possible recognition by a T cell. A step forward in this sense has been taken by Jørgensen and Rasmussen, who respectively developed NetMHCstab and NetMHCstabpan, which are ANN-based methods for predicting the stability of the pHLA complex [197,198]. They have shown that the integration of peptide-HLA binding affinity predictors with pHLA stability prediction significantly improves the identification of CTL epitopes. Moreover, assuming that longer lasting epitope presentation increases the likelihood of T cell recognition, they show that pMHC stability better correlates with immunogenicity than HLA binding affinity.

#### 4.2.2. Inherent Peptide Immunogenicity

Immunogenicity is defined as the ability of a given antigen—in this case, a pHLA complex—to elicit an immune response. There are different possible approaches to address this problem. One of these considers immunogenicity as an inherent property of the peptide. The first attempts to predict immunogenicity produced POPI (an SVM-based predictor) and POPSIK (an SVM-based method using the weighted degree string kernel). These models explored the physicochemical properties derived by the AA index [199] of the amino acid composing the peptides. Of the properties selected by the models, four were hydrophobicity-related and two were residue volume-related [200,201].

Calis et al. produced a scoring model derived by summing the log enrichment scores of amino acids found at non-anchor positions weighted by the importance of that position for immunogenic peptides compared to non-immunogenic peptides [202]. In the same year, Saethang et al. developed a method for predicting T cell reactivity based on peptide encoding using a combination of amino acid pairwise contact potentials (AAPPs) and quantum topological molecular similarity (QTMS) descriptors [203]. More recently, Zhang et al. attempted to predict immunogenic peptides based on their sequences. They analyzed the relationships between different features vis-à-vis immunogenicity and selected the optimal feature subset using a genetic algorithm [204].

Some interesting attempts to produce an integrated method are NepTepi [205], which comprises several of the HLA-binding affinity methods described above, the pMHC complex stability NetMHCStab, and the immunogenicity model produced by Calis et al.

Despite these efforts, however, immunogenicity remains a feature that is far more difficult to model and predict than mere MHC-binding affinity. On one hand, this might be a problem that is just due to the limited amount of data. On the other hand, it might reflect a more complex biological scenario; for example, genetic background (HLA haplotype), central tolerance, and TCR repertoire.

#### 4.2.3. Interaction with T Cells

All existing approaches to predicting epitope presentation do not consider whether that epitope is going to be recognized by a CTL. Previously, it has been shown how immunogenicity can, to a certain extent, be predicted by analyzing the sequence-specific features of HLA-bound peptides. Nevertheless, the results have been poor, as only one side of the immunological synapsis has been considered. Recently, the more widely used multimers-based technology [206] and the development of single cell TCR sequencing have, to a certain extent, filled the gap between TCR sequences and their recognition specificity, thus increasing the amount of experimental data available. This has given rise to different databases, such as McPAS-TCR [207], VDJdb [208,209], IEDB [210], STCRDab [211], and ATLAS [212].

An interesting work that brings peptide–TCR recognition to the same side is that of Gielis et al., who identified epitope-specific TCR sequences using a random forest algorithm [213]. However, it can only predict TCR sequences based on the peptides present in the training database.

Oghishi and Yotsuyanagi et al. abandoned the long-standing idea that the TCR repertoire is highly stochastic and individualized, showing that epitope recognition by T cells is to some extent predictable. The authors used a method to approximate the molecular scanning process of the presented peptides by the TCR repertoire, reducing the problem to an alignment-based approach [214].

Another valuable strategy for the future is to undertake structural modeling of the interaction between the pMHC and the TCR. With this concept in mind, Jensen et al. developed the TCRp-MHC model [215], a tool that accepts as input the amino acid sequence of both the peptide and the TCR of interest (alpha and beta chains). The tool automatically identifies the best structural templates and generates a structural model of the target using comparative modeling. 

TCRp-MHC models the pMHC and the TCR separately using MODELLER and LYRA [216], respectively, after which they are assembled in an additional modeling step to form the full TCR-pMHC complex. Interestingly, the structural models generated by the tool have high quality and are generated within a computational time of only 2 min.

## 5. Conclusions

The advancements in antigen discovery have paved the way for a deeper understating of the complex interaction between peptides and the HLA-I complex on one side and between HLA-I-restricted peptides and CD8+ T cells on the other. This knowledge is the key to developing efficient strategies for cancer therapeutic vaccines. To reach a better outcome and to exploit the overall potential of the antigen discovery approaches, several challenges must be addressed. One is that the HLA in vitro methods suffer from a lack of lab-to-lab reproducibility, hampering the impartial comparison of the generated HLA-I ligand datasets. This issue demands the urgent standardization of the protocol used for the isolation of peptides. Moreover, the community would generally benefit from technical advancements that allow the analysis of small amounts of tumor material (e.g., needle biopsies). In addition, the availability of open access datasets encourages collaboration among the different labs involved in antigen discovery. This will ultimately facilitate reliable progress in the field of cancer immunotherapeutic treatment. Regarding the in silico approaches, the production of an increasing amount of experimental data surely facilitates the generation of models to predict given phenomena. However, the presence of sparse databases which sometimes contain redundant data makes the collection, processing, and analysis of data tedious and difficult. The continuous production of new prediction algorithms is a great achievement; however, benchmarking these tools is an increasingly difficult task because of the limited amount of data in the respective training sets. Moreover, the use of too many different tools and pipelines creates confusion about the best practices to follow.

We believe that the combination of in vitro methods and in silico approaches is the key to tackling the complex interaction between tumor antigens and the T cell immune response. This necessary interdisciplinary expertise would expand our knowledge and ultimately benefit cancer patients.
